# A COVID-19 patient with multiple negative results for PCR assays outside Wuhan, China: a case report

**DOI:** 10.1186/s12879-020-05245-7

**Published:** 2020-07-16

**Authors:** Li-Da Chen, Hao Li, Yu-Ming Ye, Zhi Wu, Ya-Ping Huang, Wei-Liang Zhang, Li Lin

**Affiliations:** Department of Respiratory and Critical Care Medicine, Zhangzhou Affiliated Hospital of Fujian Medical University, Address: No 59, Shenglixi road, Xiangcheng district, Zhangzhou, Fujian province People’s Republic of China 363000

**Keywords:** Coronavirus disease 2019, severe acute respiratory syndrome coronavirus 2, Real-time RT-PCR, False negative

## Abstract

**Background:**

The outbreak of coronavirus disease 2019 (COVID-19) caused by severe acute respiratory syndrome coronavirus 2 (SARS-CoV-2) has become a public health emergency of major international concern. Real-time RT-PCR assays are recommended for diagnosis of COVID-19. Here we report a rare case of COVID-19 with multiple negative results for PCR assays outside Wuhan, China.

**Case presentation:**

A 32-year old male was admitted to our hospital because of 6 days of unexplained fever on January 29, 2020. He had come from Wuhan city 10 days before admission. Five days before admission, no abnormality was noted in laboratory test, chest radiography, and nasopharyngeal swab test for the SARS-CoV-2 nucleic acid. The patient was treated with ibuprofen for alleviating fever. On admission, chest computed tomography showed multiple ground-glass opacities in right lower lung field. COVID-19 was suspected. Three times of nasopharyngeal swab specimens were collected after admission. However, none of the specimens were positive. The patient was confirmed with COVID-19 after fifth SARS-CoV-2 nucleic acid test. He was treated with lopinavir/ritonavir, recombinant human interferon alfa-2b inhalation, methylprednisolone. After 18 days of treatment, he was discharged with improved symptoms, lung lesions and negative results of nasopharyngeal swab.

**Conclusion:**

This case reminds clinician that a patient with high clinical suspicion of COVID-19 but multiple negative RT-PCR result should not be taken out of isolation. A combination of patient’s exposure history, clinical manifestations, laboratory tests, and typical imaging findings plays a vital role in making preliminary diagnosis and guide early isolation and treatment. Repeat swab tests are helpful in diagnosis for this kind of patients.

## Background

In December, 2019, a new type of pneumonia caused by the severe acute respiratory syndrome coronavirus 2 (SARS-CoV-2) broke out in Wuhan City, China. The disease has been officially named coronavirus disease 2019 (COVID-19) by World Health Organization (WHO). Human-to-human transmission is the main way of causing infection [1]. WHO has declared the new outbreak to be a public health emergency of international concern. Real-time RT-PCR assays are recommended for diagnosis of COVID-19. However, the false negative PCR result has been attracted significant attention recently. We herein report a case of COVID-19 with multiple negative results for PCR assays outside Wuhan city.

## Case presentation

**Fig. 1:**
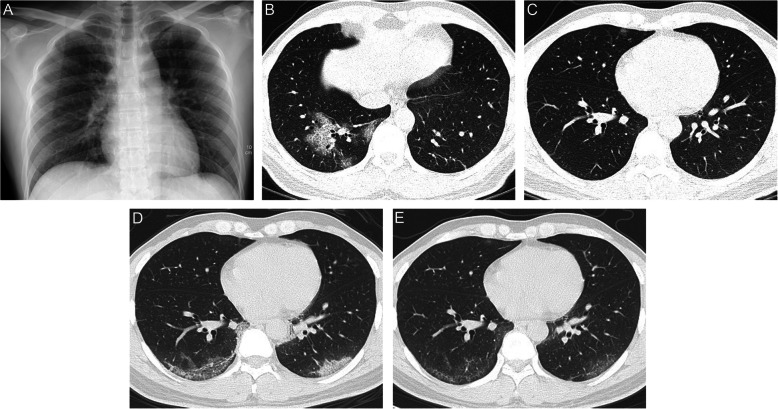
Imaging findings of the patient. **a** CXR showed no abnormality 5 days before admission; **b** and **c** Chest CT showed multiple GGOs in right lower lung field (admission day); **d** Repeat chest CT displayed larger areas of GGOs in both lower lung with a pronounced peripheral distribution imaging (day 8); **e** Repeat chest CT showed remission of lung lesions, with reduced density of GGOs (day 17). Abbreviation: CXR = chest X-ray, CT = computed tomography, GGOs = ground-glass opacities

A 32-year old male was admitted to our hospital because of 6 days of unexplained fever on January 29, 2020. The patient’s chief complaint was fever, nasal congestion, rhinorrhea, fatigue, and myalgia. He had no cough, hemoptysis, headache, sore throat, shortness of breath, nausea or diarrhea. Five days before this admission, the patient presented to fever clinics with a fever. Leukocyte count and lymphocyte count were within normal range. No abnormality was noted in Chest Radiography (CXR) (Fig. 1a). The initial nasopharyngeal swab test for the SARS-CoV-2 nucleic acid (real-time RT-PCR Kit provided by Shanghai ZJ Bio-Tech Co, Ltd., Shanghai, China) was negative. The patient was treated with ibuprofen for alleviating fever. Two days before this admission, he still had a fever (up to a maximum of 39.0 °C). He had no underlying medical conditions and was in general good health. He disclosed that he had arrived at Zhangzhou on January 19, 2020 from Wuhan city.

The patient’s physical findings on admission were as follows: body temperature, 38.4 °C; respiratory rate, 22 breaths/min; blood pressure, 124/82 mmHg; pulse rate, 113 beats/min. Physical examination of the lungs was normal. His laboratory findings on admission revealed leukopenia, lymphopenia, and mild liver injury (Table 1). Chest computed tomography (CT) showed multiple ground-glass opacities (GGOs) in right lower lung field (Fig. 1b and Fig. 1c). Nasopharyngeal swab specimens were collected on January 29, 30 and February 1 for SARS-CoV-2 nucleic acid test (real-time RT-PCR Kit provided by Shanghai ZJ Bio-Tech Co, Ltd., Shanghai, China), however, none of the specimens were positive.

**Table 1:** Laboratory findings and SARS-CoV-2 nucleic acid results

		Leukocyte count, cells/mm^3^	Lymphocyte count, cells/mm^3^	Creatinine, umol/L	ALT, U/L	AST, U/L	GGT,U/L	Hs-CRP, mg/L	SARS-CoV-2 nucleic acid assay
Reference Range		4000–10,000	800–4000	40–133	5–42	1–42	5–40	0–3	–
January 24	5 days before admission	8150	1580	–	–	–	–	34.1	Negative
January 29	Admission day	3760	1270	82.8	46.4	31.6	45	–	Negative
January 30	Day 2	3170	690	–	–	–	–	23.7	Negative
February 1	Day 4	–	–	–	–	–	–	–	Negative
February 2	Day 5	–	–	–	–	–	–	–	Positive
February 4	Day 7	8320	1500	75.9	26	20.9	32.2	31.9	–
February 7	Day 10	–	–	–	–	–	–	–	Negative
February 8	Day 11	6600	1410	60.4	29.3	29.8	29.0	3.07	–
February 10	Day 13	–	–	–	–	–	–	–	Negative
February 12	Day 15	6020	3240	–	–	–	–	–	–
February 13	Day 16	–	–	–	–	–	–	–	Negative

Abbreviation: *ALT* alanine aminotransferase, *AST* aspartate aminotransferase, *GGT* γ-Glutamyl transpeptidase, *hs-CRP* high-sensitivity C-reactive protein, *SARS-CoV-2* severe acute respiratory syndrome coronavirus 2

Given the patient’s travel history, clinical manifestations, imaging characteristics, and laboratory tests, COVID-19 was suspected and treatment with lopinavir/ritonavir, recombinant human interferon alfa-2b inhalation, methylprednisolone commenced. Another nasopharyngeal swab test was performed on February 2. Finally, the Centers for Disease Control confirmed that the fifth SARS-CoV-2 nucleic acid assay (real-time RT-PCR Kit provided by Shanghai ZJ Bio-Tech Co., Ltd., Shanghai, China and confirmed by another Kit provided by Jiangsu Bioperfectus Technologies Co., Ltd., Jiangsu, China) was positive and the diagnosis of Covid-19 was established according to the diagnostic criteria in China (General Office of National Health Commission, 2020). The procedure of real-time RT-PCR Kit was showed in Additional file 1.

On days 2 through 5 of hospitalization, his vital signs remained stable with improvement of symptoms including nasal congestion, rhinorrhea, fatigue, myalgia, and fever. The patient had no fever since day 6. He became free of symptoms afterward. On day 5 of treatment, methylprednisolone was withdrawn. From day 7, his laboratory results including liver enzymes and leukocyte count, lymphocyte count got better improvement and then kept stable until entire treatment (Table 1). The repeat CT imaging (day 8) displayed larger areas of GGOs in both lower lungs with a pronounced peripheral distribution (Fig. 1d). Given a progression of pneumonia evidenced by chest imaging, arbidol was administrated. Repeat chest CT was performed, which showed that both lung lesions diminished on the day 17 (Fig. 1e). According to the persistent negative results of SARS-CoV-2 on day10, 13, and 16, as well as the lung lesions diminished, the patient was discharged on day 18.

## Discussion and conclusion

The main symptoms of the patient were fever, nasal congestion, rhinorrhea, fatigue, and myalgia, which are common to any acute respiratory virus infection. Guan et al. [2] analyzed data of 1099 COVID-19 patients and found that fever (88.7% during hospitalization) and cough (67.8%) were the most common symptoms. The proportion of other symptoms including nasal congestion, myalgia, and fatigue are 4.8, 14.9, 38.1%, respectively. However, cough and sputum production were absent in our case. The patient’s laboratory result revealed leukopenia, lymphopenia, and mild liver injury. Guan et al. [2] reported that 21.3% of the included patients had leukopenia, 83.2% had lymphopenia, and 33.7% had elevation of alanine aminotransferase. The high prevalence of lymphopenia indicates that it may be a useful maker of COVID-19.

Previous study has concluded some typical chest CT imaging features of the COVID-19 pneumonia [3]. This includes bilateral, multifocal ground glass opacities, peripheral distribution, frequently multilobar involvement and more frequent in the lower lobes. The imaging findings of our case are in accord with the listed characteristics above. Another phenomenon worth to be mentioned is that the improvement of symptoms (day 6) and laboratory results (day 7) were 1 week earlier than the improvement of imaging findings (day 17). Therefore, improvement of symptoms and laboratory test could serve as an early marker to predict clinical outcome.

Due to the limitations of sample collection and transportation and kit performance, the positive rate of throat swab tested by RT-PCR was about 30–60% [4]. Clinicians have noted that some cases with positive chest CT findings might present with negative RT-PCR results. A study including 1014 patients suspected of COVID-19 reported that there were 413 patients with negative RT-PCR result, of 413, 308 patients had positive chest CT findings; of 308, 48% were considered as highly likely cases, with 33% as probable cases [5]. Another study reported a group of 5 patients with typical imaging findings suggesting COVID-19 had negative RT-PCR results initially. They were confirmed with COVID-19 by second or third times of swab tests [6]. In the present case, the patient was confirmed with COVID-19 after fifth times of swab tests. Since the false negative result could result in delayed treatment and increased risk of spread of COVID-19 in community or general ward. The present case reminds clinicians that a patient with high clinical suspicion of COVID-19 but multiple negative RT-PCR result should not be taken out of isolation. Repeated swab tests are helpful to make a confirmed diagnosis in this kind of patients.

Several reasons may account for the false negative RT-PCR results. First, the body viral load is a vital factor affecting detection. Viral load is associated with disease severity and disease course. A study found that the viral loads in throat swab and sputum samples peaked at around 5–6 days after symptom onset [7]. Second, viral load varies in different samples. It is suggested sputum samples had higher viral loads than throat swab samples [7]. Third, the extracted sample should contain sufficient cellular material for detection. A standardized sample collection is of importance. Finally, other factors such as kit performance, sample transportation, sample storage condition, standardized operation, results interpretation, and quality control can affect test results [8].

In summary, we report a rare case of COVID-19 with multiple negative results for RT-PCR assays. This report emphasizes that a combination of patient’s exposure history, clinical manifestations, laboratory tests, and typical imaging findings plays a vital role in making preliminary diagnosis and guide early isolation and treatment. A patient with high clinical suspicion of COVID-19 but multiple negative RT-PCR result should not be taken out of isolation. Repeat swab tests may be useful for the diagnosis for patients with an initially negative RT-PCR result.

## Supplementary information

**Additional file 1.** Procedure of real-time RT-PCR Kit.

## Data Availability

All data generated or analyzed during this study are included in this published article.

## Abbreviations

COVID-19: Coronavirus disease 2019
SARS-CoV-2: Severe Acute Respiratory Syndrome Coronavirus 2
CXR: Chest X-ray
CT: Computed tomography
GGOs: Ground-glass opacities
WHO: World Health Organization.
