# Supplementary material for: A COVID-19 patient with multiple negative results for PCR assays outside Wuhan, China: a case report
Source: BMC Infect Dis. 2020 Jul 16;20:517. doi: 10.1186/s12879-020-05245-7 (PMC7364136; doi:10.1186/s12879-020-05245-7)
Supplement: Supplementary file 1 — Additional file 1. Procedure of real-time RT-PCR Kit. [file 12879_2020_5245_MOESM1_ESM.pdf]

The procedure of real-time RT-PCR Kit:

RT-PCR assays were performed using the real-time RT-PCR Kit (Shanghai ZJ Bio-Tech Co, Ltd, Shanghai, China), targeting the open reading frame lab (ORF1ab) and nucleoprotein (N) gene regions. The PCR parameters were 45 ℃ for 10 min, 95 ℃ for 3 min, followed by 45 cycles of 95 ℃ for 15 s, 58 ℃ for 30 s, and a single fluorescence detection point at 58℃. When two targets tested positive by specific real-time RT-PCR, the case would be considered to be positive. A cycle threshold value (Ct-value) less than 37 was defined as a positive test, and a Ct-value of 40 or more was defined as a negative test. A medium load, defined as a Ct-value of 37 to less than 40, required confirmation by retesting. If the sample was tested positive using the kit provided by Shanghai ZJ Bio-Tech Co, Ltd, Shanghai, China, it was retested using a real-time RT-PCR kit provided by Jiangsu Bioperfectus Technologies Co., Ltd, Jiangsu, China. Only both were positive, case can be considered to be laboratory-confirmed.
